# Dose-Dependent Effects of Protocatechuic Acid on Motility, Redox Balance, and DNA Integrity of Frozen–Thawed Ram Spermatozoa

**DOI:** 10.3390/ijms27156817

**Published:** 2026-07-29

**Authors:** Umut Taşdemir, Fatih Avdatek, Muhammed Enes İnanç, Şükrü Güngör, Mehmet Fuat Gülhan, Barış Denk, Murat Kırıkkulak, Deniz Yeni

**Affiliations:** 1 Department of Reproduction and Artificial Insemination, Faculty of Veterinary Medicine, Ankara University, Ankara 06070, Türkiye; 2 Department of Reproduction and Artificial Insemination, Faculty of Veterinary Medicine, Afyon Kocatepe University, Afyonkarahisar 03200, Türkiye; 3 Department of Reproduction and Artificial Insemination, Faculty of Veterinary Medicine, Burdur Mehmet Akif Ersoy University, Burdur 15030, Türkiye; 4 Department of Medicinal Aromatic Plants, Technical Sciences Vocational School, Aksaray University, Aksaray 68100, Türkiye; 5 Department of Biochemistry, Faculty of Veterinary Medicine, Afyon Kocatepe University, Afyonkarahisar 03200, Türkiye

**Keywords:** protocatechuic acid, ram semen, cryopreservation, oxidative stress, DNA integrity, sperm motility, antioxidant

## Abstract

This study investigated the effects of supplementing Tris semen extender with protocatechuic acid (PCA) at doses of 25, 50, and 100 µg/mL on the quality of ram spermatozoa after cryopreservation. We evaluated motility, kinematic parameters, DNA integrity, redox status, plasma membrane–acrosome integrity (PMAI), viability, and high mitochondrial membrane potential (HMMP). PCA supplementation produced concentration-dependent and parameter-specific responses. The 25 µg/mL PCA group exhibited the most favorable functional profile, demonstrating improved progressive and total motility alongside reduced DNA damage. This protective effect was evidenced by lower tail length, tail DNA percentage, and tail moment in the comet assay. Conversely, treatment with 100 µg/mL PCA appeared to exert a notable effect on the redox balance, indicated by lower malondialdehyde, total oxidant status, and oxidative stress index levels, alongside an elevated total antioxidant status (*p* < 0.05). However, this higher concentration (100 µg/mL) did not provide parallel DNA protection and was associated with increased comet assay indices. PMAI, viability, and HMMP were not significantly affected by PCA supplementation (*p* > 0.05). These findings indicate that PCA exerts dose-dependent but parameter-specific effects on frozen–thawed ram spermatozoa. Low-dose PCA, particularly 25 µg/mL, appears more suitable for preserving motility and nuclear integrity, whereas 100 µg/mL mainly improves oxidative balance.

## 1. Introduction

Semen cryopreservation is an essential technique in sheep breeding and conservation programs, enabling the preservation and widespread utilization of genetic material from superior rams in artificial insemination (AI) programs [1]. However, the efficacy of this technique in small ruminants is often limited by reduced post-thaw semen quality. This decline is primarily driven by cryoinjury mechanisms, including osmotic stress, intracellular ice formation, membrane damage, and the excessive generation of reactive oxygen species (ROS) [2]. In sheep, pregnancy outcomes after artificial insemination with frozen semen are influenced by post-thaw semen quality traits, including sperm concentration, motility parameters, abnormal morphology, and the proportion of viable acrosome-intact spermatozoa [3]. However, individual in vitro sperm function tests may not consistently predict ram fertility, as motility, viability, acrosomal status, capacitation-related parameters, plasma membrane stability, and osmotic resistance have not always shown reliable associations with in vivo fertility [4]. Therefore, AI success should be interpreted as a multifactorial outcome involving both semen quality and non-semen-related factors [3,5]. Accordingly, studies aimed at improving the success of ram semen cryopreservation should focus not only on motility but also on the combined preservation of the structural, bio-chemical, and functional integrity of the sperm cell.

Oxidative stress is one of the major contributors to cryopreservation-induced sperm damage, as it reflects an imbalance in intracellular oxidation–reduction reactions and can compromise the structural and functional integrity of spermatozoa. During the freeze–thaw process, the restoration of the extracellular microenvironment and metabolic activity can trigger a rapid surge in reactive oxygen species (ROS). Because spermatozoa possess limited cytoplasmic antioxidant reserves and lack the capacity to mount a genomic antioxidant response, this uncontrolled ROS accumulation severely impairs sperm function [6]. Low and controlled ROS levels are required for physiological sperm functions, including motility, capacitation, hyperactivation, and the acrosome reaction [7]. However, excessive ROS accumulation can impair sperm function by inducing lipid peroxidation, protein oxidation, mitochondrial dysfunction, and DNA fragmentation, thereby compromising membrane integrity and genetic stability [8]. Ram spermatozoa are particularly vulnerable to oxidative damage during cryopreservation because their plasma membranes contain high levels of polyunsaturated fatty acids, which are prone to lipid peroxidation, whereas their cytoplasmic antioxidant defenses are relatively limited [9]. Post-thaw sperm quality cannot be adequately assessed by motility alone, because cryoinjury may affect sperm movement, DNA integrity, plasma membrane–acrosome integrity (PMAI), viability, mitochondrial function, and oxidative status at different levels [10]. Therefore, a multidimensional evaluation approach that includes motility, kinematic characteristics, redox markers, DNA integrity, and flow cytometric parameters is required to determine the true cryoprotective value of an antioxidant additive [11]. In recent years, the supplementation of semen extenders with antioxidants has emerged as an important strategy to reduce oxidative stress-related sperm damage and improve sperm quality after preservation [12]. Antioxidant-based strategies are particularly relevant in small-ruminant semen cryopreservation because oxidative stress is a key molecular mechanism contributing to cryodamage and impaired post-thaw sperm function in these species [9]. Phenolic compounds may exhibit protective antioxidant potential because their aromatic structures and hydroxyl groups enable free-radical stabilization or scavenging [13]. In addition, polyphenols can act as metal chelators, and Fe^2+^ chelation may reduce Fenton reaction-mediated oxidative damage; they may also interact with endogenous antioxidant pathways and support antioxidant defense mechanisms [14]. Nevertheless, the efficacy of antioxidant additives in semen preservation should be interpreted in a species-specific context, and findings from other animal models should not be directly extrapolated to ram semen cryopreservation [15]. Moreover, antioxidant responses may vary according to concentration and the sperm quality endpoint evaluated, as shown by the dose-dependent effects on motility, DNA integrity, flow cytometric parameters, and oxidative status in frozen–thawed ram semen [11].

Protocatechuic acid (PCA; 3,4-dihydroxybenzoic acid) is a naturally occurring, small, non-glycosylated hydroxybenzoic acid that contains an ortho-dihydroxyl (catechol) ring and a carboxyl group. Its adjacent hydroxyl groups support hydrogen- or electron-donation and stabilization of phenoxyl radicals, whereas the carboxyl group contributes to molecular polarity and behavior in aqueous biological environments [16]. This structural profile provides a specific rationale for testing PCA at the aqueous extender–sperm interface and suggests that its effective concentration range and cellular protection pattern may differ from those of larger flavonoid or stilbene antioxidants. PCA has also been associated with free-radical scavenging and the support of endogenous antioxidant defenses [17]. In male reproductive models, PCA reduced heat stress-induced testicular damage and supported the testicular antioxidant system [18], whereas dietary PCA supplementation improved boar semen quality through redox- and mitochondria-related responses [19].

PCA is structurally and functionally distinct from the antioxidants previously evaluated in ram semen preservation. Baicalein and rutin possess larger flavonoid scaffolds, chlorogenic acid is a caffeoylquinic acid ester, quinic acid lacks the aromatic catechol ring of PCA, and resveratrol is a stilbene derivative. These differences may influence solubility, membrane partitioning, cellular accessibility, and the balance between extracellular redox regulation and protection of nuclear, membrane, or mitochondrial targets. Consistent with this premise, 0.5 mM baicalein primarily preserved progressive motility and DNA integrity in frozen–thawed ram spermatozoa [10], whereas rutin produced a broader response involving selected kinematic variables, membrane integrity, and mitochondrial activity [20]. Chlorogenic acid improved motility, antioxidant capacity, ATP content, and membrane–acrosome integrity in chilled ram sperm [21]. Quinic acid produced a concentration-dependent response in frozen–thawed ram semen, with 100 µg/mL providing the most favorable overall profile and the higher concentration showing less favorable redox and DNA outcomes [11]. Resveratrol at 50 µM supported sperm quality, mitochondrial function, ATP content, and SIRT1/AMPK-related responses, whereas 100 µM produced adverse functional effects [22]. Therefore, the response to PCA cannot be inferred from the general antioxidant capacity of these compounds or from their effective doses, because antioxidant action in sperm preservation is compound-, concentration-, preservation-protocol-, and endpoint-dependent.

Despite the evidence obtained in testicular and dietary PCA models [18,19], the direct, short-term interaction of purified PCA with ram spermatozoa in a freezing extender has not been comprehensively characterized. To the best of our knowledge, this is the first study to directly supplement a ram semen freezing extender with PCA and simultaneously determine its concentration-dependent effects using CASA-derived motility and kinematic variables, comet-assay indices of DNA damage, MDA/TAS/TOS/OSI measurements, and flow-cytometric assessments of PMAI, viability, and HMMP. We tested the mechanistic hypothesis that the catechol-based redox activity of PCA would limit the freeze–thaw-induced oxidative burden, reflected by lower MDA, TOS, and OSI and higher TAS, and that this redox modulation would support sperm motility and DNA integrity. We further hypothesized that the response would be non-linear and cellular-compartment-specific; therefore, an improvement in bulk redox status would not necessarily be accompanied by equivalent preservation of membrane–acrosome integrity, viability, mitochondrial membrane potential, and nuclear DNA at every concentration. The genuine novelty of the study thus lies not only in testing PCA as a new extender additive, but also in determining whether PCA-induced redox changes translate concordantly across movement-related, nuclear, membrane–acrosomal, and mitochondrial endpoints.

## 2. Results

### 2.1. Sperm Motility and Kinematic Parameters

PCA supplementation significantly affected some post-thaw sperm motility parameters. Both progressive and total motility were markedly higher in the 25 µg/mL (PCA1) group (38.98 ± 2.95% and 75.29 ± 3.27%, respectively, Table 1) compared to the control (29.84 ± 2.73% and 58.33 ± 5.11%, Table 1) (*p* < 0.05), whereas these parameters remained at intermediate levels in the 50 µg/mL (PCA2) and 100 µg/mL (PCA3) groups. The percentage of slow spermatozoa was also significantly affected by PCA supplementation (*p* < 0.05). This parameter increased particularly in the medium-dose PCA group (33.21 ± 2.40%, Table 1) compared with the control group (19.50 ± 2.43%, Table 1) (*p* < 0.05). In contrast, no significant differences were detected among the groups in the percentages of rapid, medium, rapid progressive, or medium progressive spermatozoa (Table 1, *p* > 0.05). Among the kinematic parameters, VCL was significantly influenced by PCA treatment (Table 1, *p* < 0.05). VCL increased in the low dose (63.90 ± 2.28 µm/s, Table 1) and high dose (63.60 ± 1.27 µm/s, Table 1) PCA groups compared with the control group (54.49 ± 2.62 µm/s, Table 1). However, no significant differences were observed among the groups for VAP, VSL, STR, LIN, WOB, ALH, or BCF (Table 1, *p* > 0.05).

### 2.2. DNA İntegrity Parameters

The integrity of sperm DNA was profoundly affected by PCA supplementation. The PCA1 group exhibited significantly lower tail length (3.03 ± 0.05 µm vs. 9.14 ± 0.65 µm, Table 2), tail DNA content (18.27 ± 0.57% vs. 25.29 ± 1.25%, Table 2), and tail moment (4.44 ± 0.20 µm vs. 9.36 ± 0.72 µm, Table 2) compared to the control (*p* < 0.01), demonstrating a robust protective effect of low-dose PCA on chromatin stability. These findings indicate that low-dose PCA exerted a protective effect on post-thaw sperm DNA integrity. In the medium-dose PCA group (PCA2), tail length (9.70 ± 0.40 µm, Table 2) and tail DNA (25.90 ± 0.78%, Table 2) remained similar to the control group, and the tail moment (7.12 ± 0.52 µm, Table 2) did not show a clear improvement. In the high-dose PCA group (PCA3), tail length (12.53 ± 0.72 µm, Table 2) and tail DNA (31.95 ± 0.92%, Table 2) increased, whereas tail moment (8.84 ± 0.72 µm, Table 2) did not decrease significantly compared with the control group. These findings demonstrate that the effect of PCA on DNA damage was dose-dependent (*p* < 0.01).

### 2.3. Redox Parameters

Redox parameters showed a clear and dose-dependent response to PCA supplementation. MDA levels differed significantly among the groups and decreased particularly in the high-dose PCA group (70.30 ± 0.42 nmol/mL, Table 3) compared to the control (84.44 ± 1.93 nmol/mL, Table 3) (*p* < 0.01). Although MDA levels tended to decrease numerically in the low- (77.15 ± 1.27 nmol/mL, Table 3) and medium-dose (78.09 ± 1.13 nmol/mL, Table 3) PCA groups, the statistically distinct reduction was mainly evident in the high-dose PCA group. TAS increased significantly following PCA supplementation (*p* < 0.01). This increase showed a dose-related pattern, with the most pronounced elevation observed in the high-dose PCA group (1.17 ± 0.01 µmol/L, Table 3) compared to the control (0.82 ± 0.01 µmol/L, Table 3). In contrast, TOS decreased significantly after PCA treatment (*p* < 0.01), reducing from 15.80 ± 0.16 mmol/L in the control group to 12.68 ± 0.09, 11.85 ± 0.08, and 8.95 ± 0.08 mmol/L in the PCA1, PCA2, and PCA3 groups, respectively. OSI was also significantly affected by PCA supplementation (*p* < 0.01). Compared with the control group (193.99 ± 3.17, Table 3), OSI decreased in the PCA-treated groups, with the most pronounced reduction observed in the high-dose PCA group (76.82 ± 0.37, Table 3). Overall, the redox findings showed that PCA reduced oxidative stress indicators and enhanced antioxidant capacity.

### 2.4. Flow Cytometric Parameters

In the flow cytometric evaluation, PCA supplementation did not produce statistically significant changes in PMAI, viability, and HMMP. Therefore, PCA did not exert a significant effect on PMAI, viability, or HMMP in the present study (Table 4, *p* > 0.05).

## 3. Discussion

In the present study, PCA supplementation produced a clearly dose-dependent and parameter-specific response in frozen–thawed ram spermatozoa. The most balanced biological response was observed in the PCA1 group. The 25 µg/mL dose (PCA1) yielded the most balanced biological response, displaying a highly favorable profile regarding progressive motility, total motility, and DNA integrity. In contrast, PCA3 produced the greatest changes in the evaluated oxidative stress markers by decreasing MDA, TOS, and OSI values while increasing TAS levels. This finding suggests that PCA may not provide simultaneous and linear protection across all structural and functional compartments of the sperm cell; rather, the observed effects varied according to the dose used, the evaluated endpoint, and the oxidative markers assessed in the present study.

Motility analysis revealed that PCA provides superior functional support at lower concentrations. The significant increases in both progressive and total motility within the PCA1 group suggest that this specific dose effectively preserves post-thaw sperm movement capacity. A comparable protective pattern has been reported for chlorogenic acid in Hu ram semen, where 0.8 mg/mL chlorogenic acid improved progressive motility and total antioxidant capacity while reducing ROS and MDA levels [21]. Similarly, 50 µM resveratrol improved total and progressive motility, selected kinematic parameters, and antioxidant capacity in frozen–thawed ram spermatozoa [22]. These comparisons indicate that phenolic or plant-derived antioxidants may support sperm motility when used within an appropriate concentration range; however, this effect does not necessarily increase in a dose-dependent linear manner. The observation that the most prominent effect of PCA on motility occurred at the lower dose suggests that antioxidant additives may have a narrow effective dose range in cryopreservation. This interpretation is supported by Avdatek et al. [10], who reported that 0.5 mM baicalein preserved progressive motility and DNA integrity in ram spermatozoa after the freeze–thawing process. In the resveratrol study, supplementation up to 75 µM improved several post-thaw sperm quality parameters, whereas 100 µM resveratrol appeared to reduce sperm motility, HMMP, acrosome integrity, and plasma membrane integrity in frozen–thawed ram spermatozoa [22]. Therefore, the present PCA findings indicate that antioxidant efficacy is not determined solely by the antioxidant capacity of the compound but also by the applied dose and the sperm structure targeted. Regarding kinematic parameters, it is noteworthy that PCA particularly increased VCL, whereas it did not significantly affect other parameters. This suggests that PCA exerted a more selective effect on the VCL component rather than improving all kinematic aspects of sperm movement. In contrast, the present findings showed that rutin supplementation at 0.75 and 1 mM increased BCF, VSL, VAP, membrane integrity, and mitochondrial activity in ram spermatozoa [20]. Compared with the more selective effect of PCA on VCL in the present study, this evidence suggests that some flavonoid antioxidants may produce broader kinematic and functional responses, depending on compound structure, sperm source, and experimental conditions. This difference may be associated with the chemical structure of the antioxidant, sperm source, extender composition, and the ability of the compound to interact with membrane- or mitochondrial-associated targets.

The findings regarding DNA integrity represent the strongest and most significant results of the study. The decreases in tail length, tail DNA, and tail moment values in the PCA1 group indicate that low-dose PCA was able to preserve sperm DNA integrity. In contrast, the increases in tail length and tail DNA values in the PCA3 group show that, although the higher dose produced more pronounced changes in the evaluated oxidative stress markers, it did not provide the same protective profile for DNA integrity. This observation suggests that favorable changes in the measured oxidative stress markers were not consistently accompanied by equivalent protection of nuclear integrity. The beneficial effect of low-dose PCA on DNA integrity is consistent with findings showing that 0.5 mM baicalein preserved progressive motility and DNA integrity in frozen–thawed ram spermatozoa [10]. The dose-dependent effects of quinic acid on motility, DNA stability, and oxidative stress markers in frozen–thawed ram semen, with 100 µg/mL identified as the most effective concentration, further support the critical importance of the optimal dose concept in natural antioxidants [11]. In this context, the apparently stronger DNA-protective effect of PCA1 suggests that low or moderate doses of antioxidants may preserve sperm nuclear structure in a more balanced manner. The increase in DNA damage indicators at the high PCA dose can be explained within the framework of a biphasic antioxidant response. Consistent with the present results in quinic acid-treated ram semen, the 200 µg/mL dose increased DNA damage and oxidative stress indicators, suggesting that natural antioxidants may shift from protective to disruptive or pro-oxidant effects above a certain dose [11]. In frozen–thawed ram spermatozoa, the highest resveratrol concentration was associated with poorer functional outcomes, including reduced motility, HMMP, acrosome integrity, and plasma membrane integrity, and did not provide the same antioxidant advantage observed at lower concentrations [22]. Compatible with the present study, Salimi et al. [23] reported that 1 µg/mL cysteamine-loaded Se-NPs improved total motility and DNA integrity in frozen–thawed ram spermatozoa. However, higher-dose treatments showed adverse effects: 125 µg/mL cysteamine-loaded Se-NPs significantly reduced the percentage of spermatozoa with intact DNA, whereas 125 µg/mL sodium selenite increased MDA levels and impaired several sperm quality parameters. These data indicate that, for PCA to be used in cryopreservation, not only its antioxidant properties but also its dose-dependent effects on DNA integrity should be considered.

In terms of the evaluated oxidative stress markers, PCA3 produced the most pronounced response. The reductions in MDA, TOS, and OSI values, together with increased TAS levels at this dose, suggest that PCA favorably influenced the measured biochemical indicators of oxidative status within the seminal environment. However, these findings should be interpreted as reflecting changes in global oxidative stress markers rather than direct evidence of intracellular or mitochondrial ROS regulation. Coherent with these findings, chlorogenic acid, particularly at 0.8 mg/mL, reduced ROS and MDA levels while increasing CAT, SOD, ATP, and total antioxidant capacity, suggesting that phenolic acids may support sperm oxidative status when used at an appropriate concentration [21]. Astaxanthin supplementation, particularly at 3.5 µM, increased total antioxidant capacity and HMMP while reducing ROS and MDA levels in ram semen [24]. These findings suggest that some lipophilic antioxidants may influence a broader range of oxidative and functional endpoints than was observed for PCA under the present experimental conditions. Nevertheless, an important finding of the present study is that PCA3 produced the greatest improvement in the measured oxidative stress markers but did not show parallel superiority in motility, DNA integrity, PMAI, viability, or HMMP. These results indicate that favorable changes in the evaluated biochemical markers of oxidative status were not necessarily accompanied by simultaneous protection across all structural and functional compartments of the sperm cell. In other words, PCA supplementation was associated with improved values of the measured oxidative stress markers; however, comparable improvements were not consistently observed for membrane–acrosome integrity, HMMP, or nuclear DNA. When considering studies using antioxidants targeting mitochondria, this apparent dissociation between biochemical and functional outcomes becomes more understandable. MitoQ and mitochondria-targeted TEMPO supplementation in freezing extenders have been reported to preserve post-thaw sperm quality parameters and reduce apoptotic-like changes, DNA fragmentation, H_2_O_2_, and MDA levels, with the combined MitoQ + Mito-TEMPO treatment showing the most favorable overall response [25]. Similarly, supplementation with mitochondria-targeted α-phenyl-N-tert-butylnitrone, particularly at 100 and 150 µmol/L, increased total and progressive motility, membrane integrity, mitochondrial activity, viability, TAC, and GPx activity while reducing ROS levels in cryopreserved ram spermatozoa; ATP content reached its highest level in the 150 µmol/L group [26]. These findings suggest that antioxidants directly targeting mitochondrial or membrane-associated oxidative damage may translate biochemical improvements into broader functional protection more effectively than observed for PCA under the present experimental conditions.

Flow cytometric findings showed that PCA did not produce significant changes in PMAI, viability, or HMMP. This result indicates that, despite favorable changes in the measured oxidative stress markers, PCA did not provide marked protection for PMAI or HMMP. In a study where various concentrations (0.25%, 0.5%, 1%, and 2% *v*/*v*) of essential oils obtained from the peels of *Citrus sinensis*, *Citrus limon*, and *Punica granatum* were added to semen extender to reduce oxidative stress and their effects were evaluated after thawing, unlike our findings, 0.25% *v*/*v Punica granatum* peel oil showed positive results on sperm viability and membrane integrity, and similarly to our study, 0.25% *v*/*v Punica granatum* or *Citrus sinensis* peel oils were shown to reduce MDA production and prevent DNA damage [27]. The current finding differs from astaxanthin data showing that 3.5 µM astaxanthin increased sperm viability, PMAI, and HMMP while reducing ROS and MDA levels in ram semen preservation [24]. Moreover, 50 µM resveratrol increased PMAI, HMMP, ATP content, and antioxidant capacity, whereas 100 µM resveratrol appeared to reduce several functional parameters in frozen–thawed ram spermatozoa [22]. These comparisons suggest that the limited effect of PCA on flow cytometric parameters may be related to antioxidant structure, cellular target affinity, membrane interaction, or the possibility that the tested dose range was not optimal for membrane–mitochondrial protection.

The PCA response also disagrees with the low-dose selenium nanoparticle response reported by Kaya et al. [28]. In cryopreserved ram semen, 1 µg/mL SeNP supplementation significantly increased viability and plasma membrane integrity, suggesting that low-dose SeNPs may enhance post-thaw functional sperm quality. However, because no significant differences were detected in TOS in the same study, the beneficial effect of low-dose SeNPs should be interpreted mainly as an improvement in post-thaw functional sperm quality rather than as a direct change in bulk oxidative status [28]. This distinction is also relevant for the present PCA study because PCA3 produced the most favorable changes in the measured oxidative stress markers but did not generate a parallel functional improvement in flow cytometric endpoints. When the present findings are compared with previous PCA-focused male reproductive studies, they suggest that direct semen extender supplementation may produce a response different from systemic or testicular PCA models. In a mouse testicular hyperthermia model, PCA reduced heat stress-induced testicular damage and improved testicular antioxidant defense [18]. Dietary PCA supplementation has also been reported to improve boar semen quality, reduce sperm ROS levels, and support mitochondrial function [19]. In contrast, direct addition of PCA to the ram semen extender in the present study produced a more complex response: low-dose PCA supported motility and DNA integrity, whereas high-dose PCA was associated with more favorable values of the measured oxidative stress markers but did not provide parallel superiority in membrane–acrosome integrity, viability, or HMMP.

### Study Limitations

First, ejaculate from the four rams was pooled before treatment; although pooling was used to reduce individual variation, it prevented assessment of ram-specific responses to PCA and may have masked differences in cryotolerance related to male identity and seminal-plasma composition. Second, despite the inclusion of 40 ejaculates, the use of only four rams limits the generalizability of the findings to other individuals, breeds, and production conditions because inter-individual variation is a recognized determinant of sperm response to cryopreservation. Third, no in vivo fertility trial was conducted; consequently, the observed improvements in motility, DNA integrity, and redox balance cannot be interpreted as evidence of higher pregnancy or lambing rates, since conventional in vitro sperm-function tests do not consistently predict ram fertility. Fourth, fertilization, cleavage, and embryo-development outcomes were not evaluated, so the capacity of PCA-treated spermatozoa to fertilize oocytes and support early embryo development remains undetermined. Fifth, MDA, TAS, TOS, and OSI provided downstream or global information on oxidative balance, but intracellular and mitochondrial ROS were not directly quantified; therefore, the cellular source, magnitude, and kinetics of ROS modulation by PCA could not be established. Finally, molecular endpoints such as HO-1, SOD2, NQO1, AMPK/PGC-1/Nrf1 signaling, antioxidant-enzyme expression, and apoptosis-related pathways were not measured, so the mechanism underlying the dose- and compartment-specific effects of PCA remains inferential rather than directly demonstrated. Accordingly, the present findings should be interpreted as in vitro evidence of concentration- and endpoint-specific effects of PCA, rather than proof of enhanced reproductive performance or a defined molecular mechanism.

## 4. Material and Methods

### 4.1. Semen Collection and Processing

Semen samples were collected from four Ramlic rams (a Daglic × Rambouillet crossbreed), aged between 2 and 3 years, using an artificial vagina at two-day intervals. The collection was conducted at the Afyon Kocatepe University Faculty of Veterinary Medicine Education, Research, and Practice Farm in Türkiye, during the region’s breeding season. A total of 40 ejaculates were obtained (4 ejaculates per collection day over 10 days). Following collection, ejaculates meeting the minimum quality criteria volume ≥ 0.6 mL, mass activity ≥ 3+, motility ≥ 80%, and sperm concentration ≥ 2.0 × 10^9^ spermatozoa/mL were used in the study. On each collection day, ejaculates from the four rams were pooled to eliminate individual differences, resulting in 10 independent pools. These 10 pools were assigned to experimental groups following a split-sample design and served as the biological replicates (*n* = 10) for statistical analysis. Ethical approval for animal experimentation was granted by the Local Ethics Committee of Afyon Kocatepe University (Approval No.: 49533702/169; 13 March 2024). Furthermore, all animal handling and experimental procedures complied with the ARRIVE guidelines and international animal welfare recommendations. Following collection, ejaculates meeting the minimum quality criteria—volume ≥ 0.6 mL, mass activity ≥ 3+, motility ≥ 80%, and sperm concentration ≥ 2.0 × 10^9^ spermatozoa/mL—were used in the study. A Tris-based extender was prepared consisting of 3.63 g Tris, 1.82 g citric acid, and 0.5 g fructose per 100 mL of double-distilled water, supplemented with 15% egg yolk and 6% (*v*/*v*) glycerol. PCA (Lot number: C16330310, Macklin, Chine) solutions were prepared fresh on the day of use. To prepare the concentrated PCA solution, 0.005 g of PCA was first dissolved in 100 μL of dimethyl sulfoxide (DMSO), and then the solvent volume was increased to 50 mL. As a result, 0.005 g/50 mL was adjusted to be the final maximum concentration. This stock solution served as the source for all treatment groups and was not mixed directly with the raw semen. Instead, defined volumes of the PCA stock were added to the semen extender mixture to achieve the final PCA concentrations of 25, 50, and 100 µg/mL. All final concentrations were calculated based on the total post-dilution volume of semen and extender, ensuring the accurate attainment of the intended experimental doses. The pooled semen samples were divided into four equal aliquots and diluted with the Tris-based extender to establish the experimental groups: a control group (with no PCA as in the treatment groups containing equivalent volume of DMSO) and three treatment groups with PCA concentrations of 25, 50, and 100 µg/mL PCA1, PCA2, and PCA3, respectively. The diluted samples were equilibrated at 4 °C for 2 h, loaded into 0.25 mL French straws, frozen in liquid nitrogen vapor, and stored in liquid nitrogen at −196 °C. For subsequent analyses, the frozen semen straws were thawed in a water bath at 37 °C for 30 s.

### 4.2. Assessment of Sperm Motility and Kinematic Parameters

Sperm motility and kinematic parameters were analyzed using the Computer-Assisted Sperm Analysis (CASA, Microptic S.L., SCA^®^ v.4.2, Barcelona, Spain) integrated with a phase-contrast microscope (Nikon Eclipse 50i, Tokyo, Japan). From each frozen–thawed semen straw, a 10 μL aliquot was placed on a microscope slide and covered with a coverslip. Evaluations were performed under a green-filtered, negative phase-contrast microscope at 100× magnification. Sperm cells were classified according to their curvilinear velocity as slow (10–45 μm/s), medium (45–75 μm/s), and rapid (>75 μm/s). A forward progressive movement rate of ≥75% was considered indicative of progressive motility. The assessed motility and kinematic parameters included total motility (%), progressive motility (Prog M, %), rapid (%), medium (%), slow (%), rapid progressive (R Prog, %), medium progressive (M Prog, %), curvilinear velocity (VCL, μm/s), straight-line velocity (VSL, μm/s), average path velocity (VAP, μm/s), amplitude of lateral head displacement (ALH, μm), beat-cross frequency (BCF, Hz), straightness (STR, %) [(VSL/VAP) × 100], linearity (LIN, %) [(VSL/VCL) × 100], and wobble (WOB, %) [(VAP/VCL) × 100]. For each sample, motility data were obtained by analyzing a total of 400 spermatozoa across five randomly selected microscopic fields.

### 4.3. Assessment of Sperm DNA İntegrity

To assess the integrity of spermatozoon DNA, we performed the single-cell gel electrophoresis (COMET) test following the methodology outlined by [29]. After the samples underwent electrophoresis and were stained with a fluorochrome, they were examined using an CX31 microscope (Olympus, Tokyo, Japan) fitted with a fluorescence module. The extent of DNA fragmentation was quantified via the Comet Score application (TriTek, v. 2.0). Evaluation of each sample involved the random selection and scoring of 200 individual spermatozoa from six separate fields of view. Comet assay indices such as tail length (µm), tail DNA (%), and tail moment (µm) were evaluated.

### 4.4. Flow Cytometric Analysis

Flow cytometric analysis was performed using a CytoFLEX flow cytometer (Beckman Coulter, CA, USA) equipped with a 488 nm laser (50 mW) and emission filters at 525 ± 40 nm, 585 ± 42 nm, and 610 ± 20 nm. Spermatozoa were identified based on forward scatter area (FSC-A) and side scatter area (SSC-A) characteristics. Doublets were excluded using FSC-A versus forward scatter height (FSC-H) gating. Flow cytometric data were analyzed using CytExpert software (version 2.3). An aliquot of 30 µL semen was used in every parameter and diluted with phosphate-buffered saline (PBS) to 5 × 10^6^ spermatozoa/mL [30]. Plasma membrane and acrosome integrity were assessed using the FITC-PNA/propidium iodide (PI) dual staining method. Briefly, 5 µL of FITC-PNA (100 µg/mL; Sigma, St. Louis, MO, USA, L7381) and 3 µL of PI (2.99 mM; Invitrogen Molecular Probes, L7011, Carlsbad, CA, USA) were added to 492 µL of PBS. Semen samples were incubated at 37 °C for 15 min in the dark and subsequently analyzed by flow cytometry. Spermatozoa negative for PI and FITC-PNA were classified as spermatozoa with intact PMAI [10]. Mitochondrial membrane potential was evaluated using the JC-1 staining method. A total of 5 µL of JC-1 dye (0.153 mM, Invitrogen Molecular Probes, T3168) was added to 492 µL of PBS. Semen samples were incubated at 37 °C for 15 min in the dark. Following incubation, samples were analyzed by flow cytometry, and the percentage of spermatozoa exhibiting high mitochondrial membrane potential (HMMP, %) was recorded [10]. Sperm viability was determined using the SYBR-14/PI dual staining technique. Briefly, 5 µL of SYBR-14 (1:10 diluation, Invitrogen, Molecular Probes, L7011) and 3 µL of PI were added to 492 µL of PBS. Semen samples were incubated at 37 °C for 15 min in the dark. After incubation, samples were analyzed by flow cytometry, and viable spermatozoa were identified as SYBR-positive and PI-negative cells (%) [30].

### 4.5. Redox Response Analyses

Malondialdehyde (MDA) was used to quantify the degree of lipid peroxidation (LPO), as previously described [31]. The MDA concentration was assessed based on the reaction between lipid peroxides and thiobarbituric acid, followed by the measurement of absorbance at 532 nm. The concentration of MDA was determined in units of nmol/mL. Total antioxidant status (TAS) measurements were made using a colorimetric test kit (REL Assay Diagnostics, Gaziantep, Turkey). The experiment entailed the reduction of the oxidized radical 2,2-azino-bis-3-ethylbenzothiazoline-6-sulfonic acid (ABTS) through the action of the antioxidant compounds present in the samples, leading to visible alterations in color. A spectrophotometer was utilized to measure color intensity at a wavelength of 660 nm, and the outcomes were expressed in units of mmol/L. Total oxidant status (TOS) measurements were also made using a colorimetric test kit (REL Assay Diagnostics, Gaziantep, Turkey). The methodology employed in this study involved the assessment of the oxidation of Fe^2+^ to Fe^3+^ using oxidizing agents. The concentration was measured via spectrophotometric analysis at a wavelength of 660 nm and determined in μmol/L. Finally, the oxidative stress index (OSI) was calculated using the formula OSI = [(TOS)/(TAS × 100)] [32].

### 4.6. Statistical Analyses

Statistical analyses were performed using SPSS 22 software. The experimental unit was defined as the ejaculate/pool used for each treatment comparison, whereas sperm cells evaluated within each sample were considered technical observations. Data normality was assessed using the Shapiro–Wilk test, and homogeneity of variances was evaluated using Levene’s test. For normally distributed variables, one-way analysis of variance (ANOVA) was performed. When variances were homogeneous, Duncan’s multiple range test was used for post-hoc comparisons; when homogeneity of variances was not satisfied, Tamhane’s T2 test was applied. For variables that did not meet the normality assumption, non-parametric analyses were performed using the Kruskal–Wallis test followed by pairwise comparisons. Statistical significance was set at *p* < 0.05. All data across tables and figures are presented as mean ± SEM.

## 5. Conclusions

This study demonstrated that PCA supplementation exerts dose-dependent and parameter-specific effects on frozen–thawed ram spermatozoa. In particular, PCA1 exhibited the most balanced and biologically favorable profile in terms of progressive motility, total motility, and DNA integrity. In contrast, PCA3 produced a stronger improvement in redox-related parameters by decreasing MDA, TOS, and OSI levels while increasing TAS values; however, this effect did not translate into parallel superiority in DNA integrity, PMAI, viability, or HMMP. These findings indicate that PCA may be a promising natural antioxidant additive for ram semen cryopreservation, but its efficacy cannot be evaluated solely on the basis of overall antioxidant capacity or increasing dose. Therefore, low-dose PCA (25 µg/mL) holds significant promise as a targeted natural antioxidant for preserving the functional and genomic integrity of cryopreserved ram semen, whereas the higher dose primarily modulates the oxidative balance.

## Figures and Tables

**Table 1:** Sperm motility and velocity parameters in control and treated groups.

Parameters	Control	PCA1	PCA2	PCA3	*p*
**Prog. M. (%)**	29.84 ± 2.73 ^b^	38.98 ± 2.95 ^a^	31.51 ± 1.75 ^ab^	30.91 ± 2.85 ^ab^	0.049
**Motil (%)**	58.33 ± 5.11 ^b^	75.29 ± 3.27 ^a^	67.30 ± 1.44 ^ab^	68.06 ± 4.72 ^ab^	0.048
**Rapid (%)**	12.96 ± 2.44	17.90 ± 3.62	11.51 ± 2.03	14.42 ± 1.52	0.345
**Medium (%)**	25.88 ± 2.71	27.01 ± 1.91	22.58 ± 1.12	24.37 ± 2.69	0.531
**Slow (%)**	19.50 ± 2.43 ^b^	30.39 ± 3.96 ^ab^	33.21 ± 2.40 ^a^	29.27 ± 1.82 ^ab^	0.013
**R. Prog. (%)**	9.09 ± 1.32	10.45 ± 2.74	6.04 ± 0.66	8.47 ± 0.81	0.298
**M. Prog. (%)**	22.41 ± 2.35	22.41 ± 3.21	18.80 ± 1.41	22.43 ± 2.39	0.651
**VCL (µm/s)**	54.49 ± 2.62 ^b^	63.90 ± 2.28 ^a^	59.95 ± 2.45 ^ab^	63.60 ± 1.27 ^a^	0.039
**VAP (µm/s)**	43.37 ± 2.16	50.38 ± 2.42	47.71 ± 2.79	51.03 ± 1.44	0.100
**VSL (µm/s)**	34.81 ± 2.12	41.06 ± 2.78	37.94 ± 2.78	40.35 ± 1.67	0.273
**STR (%)**	74.32 ± 2.00	76.42 ± 2.40	74.56 ± 1.83	73.01 ± 1.55	0.680
**LIN (%)**	60.80 ± 2.67	61.91 ± 2.86	60.76 ± 2.56	59.73 ± 2.32	0.950
**WOB (%)**	78.02 ± 1.48	77.79 ± 1.55	78.26 ± 1.76	78.08 ± 1.59	0.997
**ALH (µm)**	1.83 ± 0.12	1.99 ± 0.06	1.90 ± 0.07	2.01 ± 0.08	0.429
**BCF (Hz)**	8.46 ± 0.32	9.51 ± 0.52	9.13 ± 0.40	8.51 ± 0.28	0.209

Comparison of sperm motility and kinematic parameters in control and experimental groups (25 µg/mL [PCA1], 50 µg/mL [PCA2], and 100 µg/mL [PCA3]). Prog. M., (progressive motility); Motil, (total motility); R. Prog., (rapid progressive); M. Prog., (medium progressive); VCL, (curvilinear velocity); VAP, (average path velocity); VSL, (straight-line velocity); STR, (straightness); LIN, (linearity); WOB, (wobble); ALH, (amplitude of lateral head displacement); BCF, (beat-cross frequency). ^a,b^ Different superscripts within the same row demonstrate significant differences.

**Table 2:** Evaluation of DNA damage in control and treated groups.

Parameters	Control	PCA1	PCA2	PCA3	*p*
**Tail Length (µm)**	9.14 ± 0.65 ^b^	3.03 ± 0.05 ^c^	9.70 ± 0.40 ^b^	12.53 ± 0.72 ^a^	0.001
**Tail DNA (%)**	25.29 ± 1.25 ^b^	18.27 ± 0.57 ^c^	25.90 ± 0.78 ^b^	31.95 ± 0.92 ^a^	0.001
**Tail Moment (µm)**	9.36 ± 0.72 ^a^	4.44 ± 0.20 ^b^	7.12 ± 0.52 ^a^	8.84 ± 0.72 ^a^	0.001

Tail length, tail DNA percentage, and tail moment were measured in control and treated groups (25 µg/mL [PCA1], 50 µg/mL [PCA2], and 100 µg/mL [PCA3]) using a comet assay. ^a,b,c^ Different superscripts within the same row demonstrate significant differences.

**Table 3:** Redox markers (MDA, TAS, TOS, and OSI) in control and treated groups.

Parameters	Control	PCA1	PCA2	PCA3	*p*
**MDA (nmol/mL)**	84.44 ± 1.93 ^a^	77.15 ± 1.27 ^a^	78.09 ± 1.13 ^a^	70.30 ± 0.42 ^b^	0.001
**TAS (mmol/L)**	0.82 ± 0.01 ^d^	0.96 ± 0.01 ^c^	1.01 ± 0.01 ^b^	1.17 ± 0.01 ^a^	0.001
**TOS (µmol/L)**	15.80 ± 0.16 ^a^	12.68 ± 0.09 ^b^	11.85 ± 0.08 ^c^	8.95 ± 0.08 ^d^	0.001
**OSI (TOS/TAS × 100)**	193.99 ± 3.17 ^a^	132.37 ± 1.22 ^ab^	117.56 ± 1.36 ^bc^	76.82 ± 0.37 ^c^	0.001

Malondialdehyde (MDA), total antioxidant status (TAS), total oxidant status (TOS), and oxidative stress index (OSI) values (mean ± SEM) in control and experimental groups (25 µg/mL [PCA1], 50 µg/mL [PCA2], and 100 µg/mL [PCA3]). For each group (25 µg/mL, 50 µg/mL, 100 µg/mL), different superscripts (a, b, c, d) within the same row demonstrate significant differences.

**Table 4:** Sperm functional parameters in control and treated groups.

Parameters	Control	PCA1	PCA2	PCA3	*p*
**PMAI (%)**	19.41 ± 3.58	19.98 ± 1.64	24.85 ± 4.54	25.99 ± 2.53	0.127
**Viability (%)**	53.34 ± 0.81	53.90 ± 3.01	51.79 ± 4.92	59.01 ± 2.35	0.189
**HMMP (%)**	26.10 ± 2.88	25.56 ± 1.34	23.50 ± 1.53	23.11 ± 2.02	0.778

Flow cytometric evaluation of plasma membrane acrosome integrity (PMAI), sperm viability (SYBR + PI-), and high mitochondrial membrane potential (HMMP) in control and experimental groups (25 µg/mL [PCA1], 50 µg/mL [PCA2], and 100 µg/mL [PCA3]).

## Data Availability

The datasets presented in this study can be found in online repositories. The names of the repository/repositories and accession number(s) can be found in the article.
